# Cognitive functioning in multiple sclerosis with focus on brainstem volume

**DOI:** 10.1007/s13760-025-02928-3

**Published:** 2025-11-03

**Authors:** Eline Van Doninck, Anna-Victoria De Keersmaecker, Marie D’hooghe, Bart Van Wijmeersch, Gaetano Perrotta, Souraya El Sankari, Vincent van Pesch, Dominique Dive, Miguel D’Haeseleer, Guy Laureys, Barbara Willekens, Lander Willem, Veronica Popescu

**Affiliations:** 1 Department of Family Medicine and Population Health, Faculty of Medicine and Health Sciences, University of Antwerp, Wilrijk, Belgium; 2 Department of Neurology, Antwerp University Hospital, Edegem, Belgium; 3 Translational Neurosciences Research Group, Faculty of Medicine and Health Sciences, University of Antwerp, Wilrijk, Belgium; 4 Department of Neurology, University Hospital Brussels, Brussels, Belgium; 5 Department of Neurology, National Multiple Sclerosis Center, Melsbroek, Belgium; 6 Center for Neurosciences, Neuroprotection and Neuromodulation Research Group, Vrije Universiteit Brussel, Brussels, Belgium; 7 University MS Center, University of Hasselt, Hasselt-Pelt, Belgium; 8 Noorderhart Hospitals, Rehabilitation & MS, Pelt, Belgium; 9 REVAL & BIOMED, Hasselt University, Hasselt, Belgium; 10 Department of Neurology, Hôpital Erasme, Université Libre de Bruxelles, Brussels, Belgium; 11 Department of Neurology, Cliniques Universitaires Saint-Luc, UCLouvain, Brussels, Belgium; 12 Department of Neurology, CHU Liège, Esneux, Belgium; 13 Faculty of Medicine and Health Sciences, University of Ghent, Ghent, Belgium; 14 Department of Neurology, University Hospital Ghent, Ghent, Belgium; 15 Laboratory of Experimental Hematology, Vaccine and Infectious Disease Institute, Faculty of Medicine and Health Sciences, University of Antwerp, Wilrijk, Belgium; 16 University of Antwerp, Center of Health Economic Research and Modelling Infectious Diseases, Wilrijk, Belgium; 17 Faculty of Medicine and Health Sciences, University of Antwerp, Universiteitsplein 1, 2610 Wilrijk, Belgium

**Keywords:** Multiple sclerosis, Atrophy, Brainstem, Cognition, BICAMS, MRI

## Abstract

**Background:**

The brainstem is a vital component of the cerebro-cerebellar network underlying cognition, however it remains unclear whether brainstem volumes are associated with cognitive functioning in MS.

**Objective:**

Investigate the relationship between brainstem volumes and cognitive impairment in MS, as assessed by the BICAMS battery (processing speed, verbal and visuospatial memory).

**Methods:**

We analyzed data from the VOLUMS (Volumetry in MS) study, including 143 MS patients. Magnetic resonance imaging (1.5/3.0 T, 3DT1-weighted images) was used for brain volumetrics and brainstem lesion counts. Cognitive data were collected using the “Brief International Assessment of Cognition for Multiple Sclerosis” (BICAMS). Correlation and stepwise logistic regression explored associations between brain volumes and cognitive performance. In a subset of 35 patients with 3-year follow-up, longitudinal changes in brain volumes and cognition were also assessed.

**Results:**

Cognitive impairment (≥ 2 standard deviations below predicted scores on at least one test) was present in 30.1% of participants. No significant correlations were found between brainstem volume and cognitive scores. Hippocampus (p = .046), thalamus (p = .024), cortex (p < .001), and gray matter (p < .001) volumes were significantly lower in cognitively impaired patients. Processing speed correlated with cortex (R = .217, p = .009) and GM (R = .206, p = .013), while verbal memory correlated with hippocampus (R = .218, p = .009), cortex (R = .251, p = .003) and GM (R = .275, p = .001) volumes. Disease duration was the only significant predictor of cognitive impairment (p < .001). In the longitudinal subset, no clear evidence of progressive volumetric decline or related cognitive deterioration was observed.

**Conclusion:**

While no link was found between brainstem volumes and cognitive impairment, this analysis underscores the importance of considering various brain structures in understanding cognitive impairment in MS.

**Supplementary Information:**

The online version contains supplementary material available at 10.1007/s13760-025-02928-3.

## Introduction

Multiple sclerosis (MS) is a common chronic neuroinflammatory and -degenerative disease that affects the central nervous system and typically has its onset in young adult age [1]. Demyelination and axonal loss are hallmarks of the disease, leading to focal lesions and atrophy [2]. The disease manifests through a range of possible symptoms, including sensory and visual disturbances, motor impairments, pain, fatigue, bowel and bladder dysfunction, and cognitive deficits. Between 30 and 65% of MS patients experience difficulties in at least one area of cognitive functioning [3], including attention, memory (working, recall, and episodic), executive functioning, and processing speed [4]. Current therapeutic strategies inadequately address these cognitive impairments, leaving a critical gap in MS management [5].

Cognitive tests are sometimes difficult and expensive to perform and are not generally available to all patients, while most patients do undergo regular brain magnetic resonance imaging (MRI). An imaging surrogate marker for cognitive impairment could help guide clinical decisions even when cognitive testing is unavailable. Whole brain volume loss is one of the most widely used imaging-based biomarkers in MS and correlates with both physical and cognitive disability. Excessive brain atrophy, exceeding the limits of normal aging at 0.5–1.35% per year, can be observed in the early stages of MS and accelerates with progression of the disease [6]. Gray matter (GM) atrophy, rather than white matter atrophy, has been identified as the primary factor contributing to disability progression [7, 8]. Cognitive impairment has been associated with GM atrophy [9–12], although the contribution of individual subcortical GM structures to cognitive dysfunctions within the pattern of atrophy is under debate. This line of research should also be considered in light of the clinico-radiological paradox, which describes the mismatch between conventional MRI markers and clinical outcomes [13]. Concepts such as cognitive reserve – the intellectual reserve that allows individuals to maintain or optimize cognitive functioning despite brain atrophy and neurodegeneration – and neuroplasticity – the compensation of neuronal damage by structural and functional reorganization in the brain – can partly explain why structural damage does not always translate into measurable impairment [14, 15]. However, research also suggests that regional volumetric and microstructural measures, rather than global volumetric measures, may provide stronger links with cognition [16]. In previous research [4], different subcortical GM structures such as the thalamus and hippocampus have been investigated. However, the contribution of brainstem atrophy to cognitive functioning remains underexplored.

The brainstem is a crucial component of the cerebro-cerebellar network that underlies cognition [17–19]. In addition to the adjacent thalamus and cerebellum, tissue loss has also been observed on MRI in the pathway-rich brainstem of MS patients [20]. In previous research, thalamic and cerebellar atrophy has been correlated with physical disability and cognitive impairment [7, 21–25]. Only one of these studies also explored correlations between brainstem atrophy and cognition using the PASAT (Paced Auditory Serial Addition Test, which assesses auditory information processing speed, flexibility and calculation ability), and found none [7]. Other studies only linked brainstem atrophy to MS-induced physical disability [26, 27]. Interestingly, isolated brainstem lesions in other neurological disorders, such as stroke, have been associated with cognitive dysfunction, manifesting as executive dysfunction, attentional deficits, a decline in general intellectual capacity and impairments in memory, language, and visuospatial skills [28, 29].

Our hypothesis is that brainstem atrophy, measured as reductions in brainstem volumes through MRI, contributes to cognitive dysfunction in MS. The aim of this study is to explore the correlation between brainstem volumes and cognitive dysfunction in individuals with MS, specifically examining performance on the BICAMS battery, which assesses processing speed, verbal memory, and visuospatial memory.

## Methods

### Study design

We used clinical and imaging data from the VOLUMS (Volumetry in MS) study, a prospective observational multi-center real-world study of the Belgian Study Group of MS (BSGMS), conducted and finalized between 2015 and 2019 with the aim of investigating the correlation between volumetric brain MRI measures and cognition in MS. The current work can be considered an exploratory extension of the original VOLUMS project, building further on its dataset but focusing on different endpoints.

### Ethical approval

This study was conducted in accordance with the Declaration of Helsinki. Approval from the ethics committee was secured on April 8th, 2015, in each participating center, with UZ Brussel serving as the primary ethics committee (B.U.N. 143201421978). Written informed consent was provided by all patients.

### Study population

Patients were recruited in 10 participating hospitals: UZ Brussel, National MS-Center Melsbroek, CHU Liege, Noorderhart Revalidatie & MS Pelt, UZ Gent, UZ Antwerpen, CHU Charleroi, Cliniques Universitaires St. Luc, ULB Erasme and CNRF de Fraiture. Inclusion criteria were diagnosis of relapsing–remitting (RRMS) or secondary progressive MS (SPMS), confirmed by the 2010 McDonald criteria [30] and age 18–60 years. Patients with primary progressive MS (PPMS) were not included, since MS medication influences brain volumes and no disease modifying treatments (DMTs) were available for PPMS at the time of the study initiation; their inclusion would therefore have introduced systematic differences in treatment exposure. There were no restrictions regarding treatment and/or Expanded Disability Status Scale (EDSS, measure of disability) at study entry. Patients with systemic, neurologic, psychiatric, or other pathologies that could affect cognition or MRI interpretation were excluded.

### Clinical data

The following demographic and clinical data were collected upon inclusion: date of birth, sex, recruiting hospital, education level (based on years of education), EDSS score, disease duration since MS diagnosis, current DMT, steroid treatment for relapse within the last 12 months, other pathologies that can influence MRI data (Alzheimer, diabetes, etc.) and cognition. To assess the different aspects of cognition, the “Brief International Assessment of Cognition for Multiple Sclerosis” (BICAMS) was used. BICAMS is a short 15-min assessment battery facilitating cognitive assessment in a feasible, consistent and valid manner. It consists of the “Symbol Digit Modality Test” (SDMT) to assess information on processing speed, the “California Verbal Learning Test-Second Edition” (CVLT-II) for verbal short-term memory and learning, and the “Brief Visuospatial Memory Test-Revised” (BVMT-R) for visual short-term memory and learning [31–33]. All tests were administered in a standardized manner across all centers, in accordance with BICAMS guidelines and using uniform instructions.

A cross-sectional analysis was performed, using baseline clinical and radiological data. In addition to raw measured cognitive scores, an expected cognition score per individual based on age, sex and education was constructed. This approach not only allows for standardized interpretation but also partly accounts for individual differences in cognitive reserve, recognizing that education is one of several factors possibly influencing this reserve [14]. The methodology for obtaining these predicted scores was derived from Costers et al. [34]. A previously published regression model was used to calculate a predicted score for each BICAMS test (Formula 1, example of formula for SDMT), accounting for age, sex and education. Regression coefficients were derived from a healthy Belgian control population, which included 97 healthy controls comparable to our MS cohort in terms of age, sex, and education (Supplement A).

Formula 1: calculation of predicted SDMT score

$$\begin{aligned}{score}_{SDMT}&={intercept}_{SDMT}+age*{\beta }_{age,SDMT}+ {age}^{2}*{\beta }_{{age}^{2}, SDMT}\\&\quad+sex*{\beta }_{sex,SDMT}+education*{\beta }_{education, SDMT}\end{aligned}$$

To align the measured scores from the VOLUMS study with the expected values, a conversion table provided by Costers et al. [34] was used to scale the measured scores (see Supplement B). This process facilitates a direct comparison between the actual test results and the demographic-based expected outcomes. To summarize, for each BICAMS test, two types of scores were generated for each participant: a raw score, as measured during the study, and an expected score reflecting the predicted outcome based on age, sex, and education. By dividing the difference between the expected score and the scaled measured score by the standard error of the residual (RSE) of the regression model, we obtain a unique delta z-score (ΔSDMT, ΔCVLT and ΔBVMT) for each individual. This z-score indicates how many standard deviations (SD) the score of an individual deviates from the expected score. Patients who scored 2 SD below the expected score on at least one test of the cognition assessment were considered cognitively impaired [35]. For an example of the workflow, see Supplement C.

### MRI acquisition and analysis

An MRI as part of routine clinical follow-up, acquired on the same day as the cognitive assessment, marked the inclusion of a certain patient (baseline MRI at year 0). A subset of the patient population also underwent a second follow-up MRI at year 3. The MRI measurements were conducted using a standardized MRI protocol (1.5 T or 3.0 T) in all participating centers to harmonize acquisition parameters. Anatomical MRI included 3D T1-weighted images (TR/TE = 2300/2.3 ms, FA = 9°, slice thickness = 1 mm, no slice gap, voxel size = 1 mm^3^, 256 × 256 mm). The processing of the cross-sectional 3D T1-weighted data at baseline was done using the “recon-all” pipeline from FreeSurfer (version 7.3.2) [36]. In addition to brainstem volumes, volumetric measurements for cortex, GM, amygdala, hippocampus, and thalamus were obtained using FreeSurfer. To derive segmentations for brainstem, amygdala, hippocampus, and thalamus, the FreeSurfers “subregion segmentation” (beta version) was used. For the subjects with assessments at two timepoints, all anatomical baseline and follow-up MRI data were processed and segmented using FreeSurfers “longitudinal stream” [37]. All segmentations were visually verified by EVD and VP was consulted for additional control in case of doubt. To correct for interindividual differences in head size, all volumes were corrected for total intracranial volume (TIV), as calculated by FreeSurfer. Based on the brainstem segmentation, brainstem lesion counts were manually assessed by VP.

### Statistical analysis

Statistical analysis was performed using SPSS (version 29.0). Visual inspection and Shapiro–Wilk tests were used to examine the normal distribution of the data. Normality criteria were not met for all measures. Descriptive statistics, t-tests, Mann–Whitney U-tests or Chi-square tests were used to analyze differences in demographics and clinical data between cognitively impaired and unimpaired patients.

In the cross-sectional analysis of the baseline data, we determined Spearman correlations between cognition scores and volumetric parameters. The correlation strength was determined using Evan’s scale [38]. Next, a stepwise logistic regression analysis was performed to assess the relative contributions of age, sex, education, disease duration, lesion count and normalized brain structure volumes in predicting cognitive impairment status. A forward stepwise analysis was conducted using the Wald statistic as the selection criterion, with P = 0.05 for entry and P = 0.10 for removal. Statistical significance was set at P < 0.05.

For the longitudinal analysis of a small subset of patients with two timepoints, volumetric changes were assessed using the Wilcoxon signed-rank test. Spearman correlations were used to explore associations between changes in brain volume and cognition as measured by SDMT over three years. The CVLT-II and BVMT-R were not included in the longitudinal analysis. A conservative approach using difference scores was chosen due to the small sample size and convergence issues in more advanced longitudinal models (e.g. linear mixed models).

## Results

### Study population

A total of 200 MS patients were recruited in the multi-centric VOLUMS study, of whom 143 patients had complete data to be included in the cross-sectional analysis at baseline (see Supplement D for detailed description of dropouts/exclusions). Steroid treatment data were available for 68% of patients in the cross-sectional analysis, and none of these had received steroids within 30 days prior to the MRI scan.

Of the 143 patients, 43 (30.1%) were classified as cognitively impaired (i.e., scoring 2 SDs below the expected score in ≥ 1 cognitive test). In total, 28 patients failed on the SDMT cognitive test (19.6%), 20 on CVLT-II (14.0%) and 15 on BVMT-R (10.5%). Demographic, clinical and volumetric MRI data for both groups can be found in Table 1. The cognitively impaired group was slightly older compared to the cognitively unimpaired group (44.6 ± 10.3y vs. 41.2 ± 9.6y) although this difference was not statistically significant (p = 0.061). However, the impaired group did show a significantly longer disease duration at inclusion (median 13 [8.0–21.0] y vs. 9[5.0–14.0] y, p = 0.002). Sex, disease type, treatment distributions, education levels, and EDSS scores at inclusion were comparable between groups. Average cognitive performance was significantly worse in the cognitively impaired group across all tests (impaired vs. unimpaired: 37.5 ± 10.3 vs. 56.2 ± 10.3, p < 0.001 for SDMT; 51.5 ± 11.2 vs. 65.3 ± 8.4, p < 0.001 for CVLT; 20.3 ± 7.3 vs. 28.5 ± 4.6, p < 0.001 for BVMT). This pattern was confirmed by the corresponding z-scores.

**Table 1:** Demographic, clinical and volumetric MRI data of patients with and without cognitive impairment

		Cognitively impaired	Cognitively unimpaired	P
Age (in years, at inclusion)		44.6 ± 10.3	41.2 ± 9.6	0.061
Sex (male/female)		13/30 (43.3%)	34/66 (51.5%)	0.660
Education level (in years)		15.0 [12.0–16.0]	15.0 [12.0–16.0]	0.479
Disease duration (in years, at inclusion)		13.0 [8.0–21.0]	9.0 [5.0–14.0]	0.002**
EDSS (at inclusion)		2.5 [2.0–4.0]	2.5 [1.5–3.5]	0.106
Disease-type	RRMS	38 (88.4%)	96 (96.0%)	0.085
	SPMS	5 (11.6%)	4 (4.0%)	
Treatment status	First-line	15 (34.9%)	51 (51%)	0.204
	Second-line	23 (53.5%)	41 (41%)	
	None	5 (11.6%)	8 (8%)	
Brainstem lesion count		2 [1–3]	2 [1–3]	0.943
Cognition scores	SDMT	37.5 ± 10.3 (N = 43)	56.2 ± 10.3 (N = 100)	< 0.001***
	CVLT-II	51.5 ± 11.2 (N = 43)	65.3 ± 8.4 (N = 98)	< 0.001***
	BVMT-R	20.3 ± 7.3 (N = 43)	28.5 ± 4.6 (N = 96)	< 0.001***
Cognition z-scores	SDMT	−2.06 ± 1.05 (N = 43)	−0.39 ± 0.96 (N = 100)	< 0.001***
	CVLT-II	−1.71 ± 1.11 (N = 43)	−0.25 ± 0.82 (N = 98)	< 0.001***
	BVMT-R	−1.17 ± 1.21 (N = 43)	0.25 ± 0.88 (N = 96)	< 0.001***
Volumes (raw, mm^3^)	Brainstem	23,113.3 [21476.5–24,413.9]	23,406.7 [21574.3–25,690.5]	0.224
	Amygdala	3212.0 [3056.1–3563.4]	3382.8 [3164.2–3653.5]	0.077
	Hippocampus	6424.0 [6086.0–7061.5]	6904.5 [6320.6–7343.1]	0.046*
	Thalamus	11,849.3 [10726.4–12,843.2]	12,590.2 [11445.9–13,604.5]	0.024*
	Cortex	419,461.7 [390181.5–453,131.5]	451,805.9 [425604.9–490,263.2]	< 0.001***
	GM	572,775.6 [536673.1–606,220.4]	615,278.0 [577452.1–656,279.7]	< 0.001***
Volumes (corrected for TIV)	Brainstem	0.01631 [0.01547–0.01877]	0.01691 [0.01549–0.01872]	0.840
	Amygdala	0.00235 [0.00218–0.00291]	0.00241 [0.00224–0.00276]	0.558
	Hippocampus	0.00459 [0.00435–0.00559]	0.00495 [0.00456–0.00550]	0.295
	Thalamus	0.00863 [0.00728–0.00965]	0.00889 [0.00797–0.01009]	0.275
	Cortex	0.30759 [0.28353–0.37052]	0.31907 [0.30260–0.36058]	0.042*
	GM	0.42153 [0.38596–0.49862]	0.43354 [0.41341–0.48162]	0.076

Independent Samples T-Test (M ± SD), Mann–Whitney U-Test (Mdn [IQR]) or Chi-Square test (%)

*P < 0.05, **P < 0.01, ***P < *0.001*, *M* = mean, *SD* = standard deviation, *Mdn* = median, *IQR* = Interquartile Range, *RRMS* = relapsing–remitting MS, *SPMS* = secondary progressive MS, *SDMT* = Symbol Digit Modalities Test, *CVLT-II* = California Verbal Learning Test, Second edition, *BVMT-R* = Brief Visuospatial Memory Test, Revised, *GM* = gray matter

No group differences were observed in brainstem lesion count (2 [1–3] for both groups, p = 0.943). All brain volumetric measures – both raw and normalized – were numerically lower in the cognitively impaired group. However, except for the normalized cortex (p = 0.042), none of the differences between normalized volumes reached statistical significance (all p > 0.05). Significant differences were observed only for raw hippocampal (p = 0.046), thalamic (p = 0.024), cortical (p < 0.001) and GM (p < 0.001) volumes, which were all significantly smaller in the cognitively impaired group.

### Cross-sectional analysis

The cross-sectional correlation analysis of the baseline data revealed weak correlations between measured SDMT scores and both cortex (R = 0.217, p = 0.009) and GM (R = 0.206, p = 0.013), in addition to weak correlations between CVLT and hippocampal (R = 0.218, p = 0.009), cortical (R = 0.251, p = 0.003), and GM volumes (R = 0.275, p = 0.001). However, none of the cognitive test results correlated significantly with brainstem volumes (all p > 0.3). Also, BVMT scores did not exhibit any significant results with any of the volumes included (all p > 0.1) (Table 2).

**Table 2:** Correlation (Spearman) between SDMT/CVLT/BVMT and the different normalized volumetric measures at baseline

		Brainstem	Amygdala	Hippocampus	Thalamus	Cortex	GM
SDMT	R	−0.003	0.087	0.127	0.109	0.217**	0.206*
	P	0.970	0.301	0.132	0.196	0.009	0.013
CVLT-II	R	0.076	0.161	0.218**	0.164	0.251**	0.275**
	P	0.372	0.057	0.009	0.053	0.003	0.001
BVMT-R	R	−0.006	0.018	0.030	−0.022	0.125	0.114
	P	0.948	0.830	0.725	0.801	0.141	0.182

*P < 0.05, **P < 0.01, ***P < *0.00, R* = Spearman correlation coefficient

A forward stepwise logistic regression identified disease duration as the only significant predictor of cognitive impairment (*p* < 0.001, OR = 1.105, 95% CI [1.043–1.170]). The model was statistically significant (χ^2^ = 12.86, *p* < 0.001) and correctly classified 75% of cases. The Hosmer–Lemeshow test indicated good model fit (*p* = 0.122). All other variables (age, sex, education, lesion count and normalized brain structure volumes) were excluded from the model. Because this was a multicenter study, we examined whether results were influenced by clustering across centers. Including center as a random intercept in a generalized linear mixed model yielded a variance estimate of 0.51 (p = 0.24), with a wide confidence interval (95% CI 0.10–2.76), indicating no significant clustering effect by center. Results therefore appear robust across centers.

### Longitudinal analysis

Of the 143 patients, data from 35 patients were ultimately included in a longitudinal analysis (see Supplement E). In the longitudinal sample, 8 patients (23%) experienced a clinically meaningful [39] decline in raw SDMT scores of more than 4 points over the three-year period. In contrast, 19 patients (54%) reported improved scores at follow-up compared to baseline. Brainstem lesion counts increased from an average of 1.4 to 1.9. Brain volume measures remained stable during the study period, with no significant changes reported (all p > 0.05). As a result, no significant associations were found between SDMT and brain volume changes over three years (all p > 0.05).

## Discussion

Our analysis of the VOLUMS data aimed to elucidate the relationship between brainstem volumes and cognitive dysfunction in MS, as measured by the BICAMS battery. While our results did not confirm the hypothesis that changes in brainstem volumes contribute to cognitive dysfunction, they do provide insights into how brain structure volumes relate to cognition performance and underscore the importance of investigating how distinct brain regions relate to different aspects of cognition in MS.

### Cross-sectional analysis

As our classification was based on the conservative criterion of performance ≤ 2 SD below the predicted score on at least one BICAMS subtest [35], the observed group differences on these same tests are expected and should not be taken as independent validation for the classification. Notably, although only one impaired score was required for classification, patients in the impaired group performed significantly worse across all three BICAMS domains (both raw scores and z-scores), supporting the robustness of this cutoff. The absence of additional independent cognitive or functional measures in our dataset can, however, be considered a limitation.

Despite an established link of brainstem volumes with motor impairment and its central position in the cerebro-cerebellar network, we found no significant correlations between brainstem volumes and cognitive test outcomes in the cross-sectional analysis. Other included brain structures (normalized hippocampus, cortex and GM), did show significant correlations with one or more of the cognition tests included in the BICAMS battery. The observed correlations of hippocampal volumes with memory and verbal learning (as assessed with the CVLT) correspond to findings reported in earlier research [40]. Significant positive correlations for both SDMT and CVLT were observed exclusively for the cortical and GM volumes. These weak correlations align with previous research [41, 42], however the lack of observed correlations between the amygdala and thalamus volumes and cognitive test performance contrasts with previous findings [40, 43, 44]. The smaller sample size and the possibility of recent steroid treatment in part of our cohort could account for the lack of correlation, as could the fact that this cohort was under DMT, including second-line treatments, which might have limited the extent of subcortical atrophy [45]. The lack of significant correlations between structural brain volumes and BVMT outcomes in our study is in line with earlier findings that have shown inconsistent or weak associations between subcortical brain regions and visuospatial memory [21, 40, 46, 47]. Our findings do show that reduced volumes in different memory- and processing-related brain regions are related to decreased cognitive function. This confirms the prevailing understanding that MS-related cognitive decline is not driven by changes in a single brain structure, but rather tied to more diffuse neurodegeneration processes and alterations in functional connectivity of GM structures [3, 18, 48, 49].

The finding that disease duration was significantly different between the impaired and unimpaired group plus the only significant predictor of cognitive impairment, suggests that longer disease duration is associated with worse cognitive functioning. While this may reflect the cumulative impact of MS pathology over time, our MRI volumetric measures did not contribute significantly to the regression analysis, and thus no direct imaging evidence of such cumulative damage was observed. Methodological considerations, discussed later, may explain why volumetric variables did not emerge as significant predictors. Nevertheless, the association with disease duration is consistent with prior work indicating that clinical progression is an important driver of cognitive decline [50].

### Longitudinal analysis

In the longitudinal subset, no significant changes in cognitive performance or brain volumes were observed over the three-year period. Correlations between volumetric changes and SDMT score changes were weak and did not reach statistical significance. These findings suggest that, within the timeframe and sample size of our study, there was no clear evidence for progressive volumetric decline or related cognitive deterioration detectable with the methods used.

### Limitations

Our findings suggest that brainstem atrophy may not play a central role in cognitive decline in MS, at least not in the way it was measured or assessed in this study. However, this interpretation must be considered in light of several biological and methodological limitations that may have influenced our ability to detect significant associations. First, the tests included in the BICAMS battery may not effectively measure cognitive functions primarily associated with the brainstem, which are attention and executive function. This misalignment might explain why brainstem volumes in MS are not related to cognitive scores derived from the BICAMS tests.

Next, methodological limitations may also have contributed to the absence of more significant findings. First, the longitudinal analysis included only 35 patients, which limited statistical power to detect subtle effects, especially in regions like the brainstem where volumetric changes are less pronounced. In addition to the different MRI setups in the participating centers, nearly 30% of follow-up scans were conducted on a different MRI scanner than at baseline. Heterogeneity in MRI configurations, both within and between centers, is a well-known confounder in longitudinal and multicenter neuroimaging studies [51] and could have obscured some genuine correlations in our data.

Furthermore, information on steroid treatment was not available for all patients included in the study. Although steroid treatment in the 30 days prior to the MRI scan and cognitive testing did not constitute a formal exclusion criterion (aiming to create a real-world dataset), none of the patients for whom the information was available had had steroid treatment in the 30 days prior to the MRI scan and cognitive testing. However, we cannot exclude that steroid treatment had been administered to some of the patients included in the dataset, and as such might have influenced the volumetric and cognitive results.

Another limitation concerns the absence of alternate SDMT forms in our study, which may have introduced practice effects. Although such effects are generally considered minimal at retest intervals exceeding two years [52], we still observed a non-significant improvement in SDMT scores over three years. This raises the question of whether the change reflects true cognitive stability or improvement, or rather the limited suitability of the SDMT for long-term monitoring. Similar apparent improvements have been described in progressive MS cohorts, where cognitive decline would be expected [52, 53]. Thus, alternate test forms should be employed whenever possible to optimize the validity of longitudinal cognitive assessments.

In addition, the absence of a healthy control group limits our ability to directly compare cognitive performance and volumetric measures with a non-MS population. While we used regression-based normative data to adjust for demographic factors and derive expected cognitive scores, inclusion of healthy controls would have strengthened the interpretability of our findings. Likewise, the exclusion of PPMS patients—due to the lack of available DMTs for this subtype at the time of the study initiation—also limits the generalizability of our results, which may not apply to patients with non-relapsing-onset forms of MS.

Also important is the fact that patient-related factors such as mood, fatigue and sleep can influence test performance on a day-to-day basis. Although these variables were not analyzed in the present study, validated self-report questionnaires are available to measure them (e.g., Beck Depression Inventory for mood, Modified Fatigue Impact Scale for fatigue). Incorporating such measures in future studies would strengthen the methodological rigor. Finally, as this was a more exploratory analysis of pre-existing data, multiple tests were not corrected for, which increases the risk of false positives and warrants cautious interpretation.

## Conclusion

In conclusion, our study highlights the need to consider the complex interplay between different brain regions when assessing cognitive decline in MS. We did not find a significant association between brainstem volumes and cognition (as measured by the BICAMS battery). So, our hypothesis driven by the crucial role of the brainstem in neural networks and its established association with physical disability in MS, was not confirmed. However, additional research with larger cohorts, cognitive tests more directly associated with brainstem functions, and healthy control groups could be interesting.

While neuroimaging-based biomarkers hold potential for predicting future decline in MS patients, the current data do not yet support their use as standalone clinical tools. However, recent publications on multimodal biomarkers in MS [53–55] underscore the importance of further research focusing on specific brain structure volumes as part of a broader biomarker framework, which may ultimately help to address not only the physical manifestations of MS but also the cognitive challenges.

## Supplementary Information

Below is the link to the electronic supplementary material.

Supplementary file1 (DOCX 1111 kb)

## Data Availability

All materials related to our paper are available from the corresponding author (EV), upon reasonable request.
