# Supplementary material for: Cognitive functioning in multiple sclerosis with focus on brainstem volume
Source: Acta Neurol Belg. 2025 Nov 3;126(1):197–206. doi: 10.1007/s13760-025-02928-3 (PMC12987810; doi:10.1007/s13760-025-02928-3)
Supplement: Supplementary file 1 — Supplementary file1 (DOCX 1111 kb) [file 13760_2025_2928_MOESM1_ESM.docx]

**Cognitive functioning in multiple sclerosis with focus on brainstem volume**

Van Doninck, E.; De Keersmaecker, A.; D’hooghe, M.; Van Wijmeersch, B.^;^ Perrotta, G; El Sankari, S; van Pesch, V.; Dive, D.; D’Haeseleer, M.; Laureys, G.; Willekens, B. Willem, L.; Popescu, V. on behalf of Belgian Study Group Multiple Sclerosis (BSGMS)

Corresponding author: Eline Van Doninck (eline.vandoninck@uantwerpen.be)

**Supplementary material**

Supplement A-1: Regression models with coefficients for the three BICAMS measures in healthy controls (N=97), as determined by Costers et al. (34)


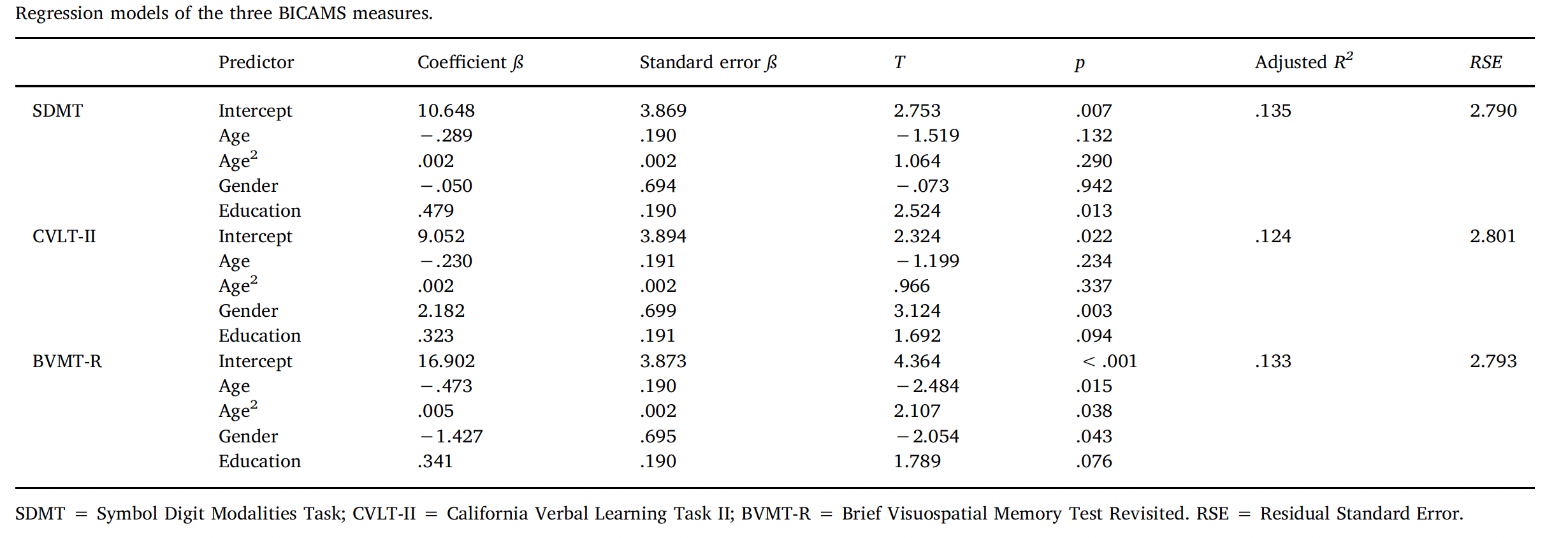


Supplement A-2: Demographics of the healthy control group from Costers et al. (34) versus the MS cohort of the VOLUMS study

| **Characteristics** | **Healthy control group (Costers et al., 2017)** | **MS cohort (VOLUMS)** |
| --- | --- | --- |
| Sample size | n = 97 | n = 143 |
| Age, mean ± SD (years) | 43.5 ± 12.7 | 42.3 ± 10.0 |
| Female (%) | 77% | 67% |
| Education, mean ± SD (years) | 14.7 ± 1.6 | 14.6 ± 2.0 |

Supplement B: Conversion table to scale observed cognition scores to normative scores, as used by Costers et al. (34)


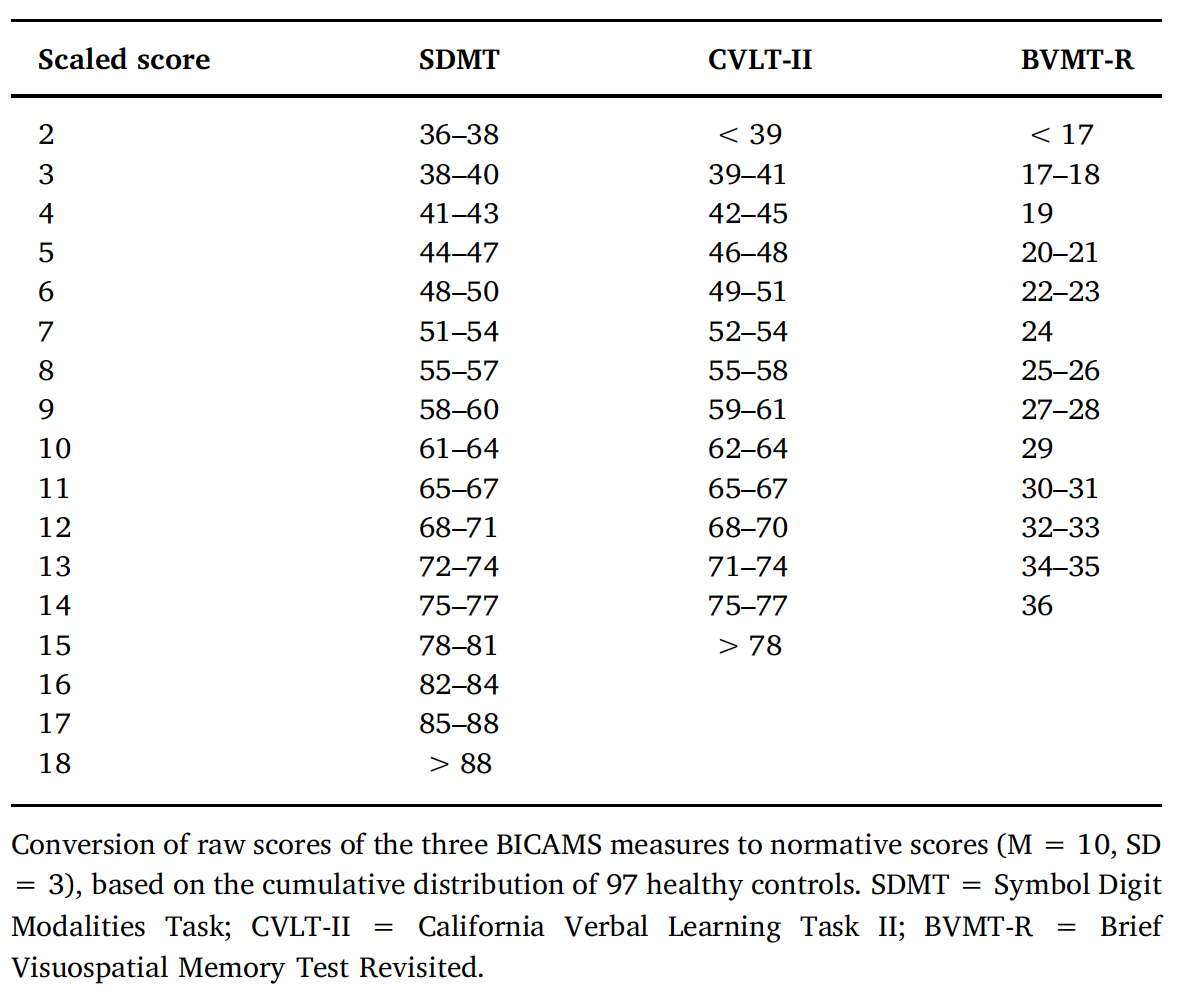


Supplement C: Example of the workflow to determine the scaled observed scores and predicted scores for one subject.

| **Subject characteristics =** | 41-year-old female (male: sex = 1; female: sex = 2) MS patient with 17 years of education, who had a score of 50 on the SDMT performed during the baseline visit. |
| --- | --- |
| **Predicted SDMT score =** | Using formula, subject characteristics and coefficients from table in Supplement A:  10.648 + 41*(-0.289) + 41^2^*(0.002) + 2*(-0.050) + 17*(0.479) = 10,20 |
| **Scaled SDMT score =** | Using conversion table in Supplement B; reported SDMT score of 50 gets scaled to 6. |
| **Δ SDMT z-score =** | (Observed/scaled score – predicted score)/RSE: (6 – 10.20) = -4.20/2.79 = 1.51 |

Supplement D: Reasons for dropouts/exclusion in different steps of the study analysis

|  | **# Patients** | **# Exclusions** | **Reason for exclusion** |
| --- | --- | --- | --- |
| **Recruited patients** | 200 |  |  |
|  |  | 8 | Discontinuation |
|  |  | 12 | Missing cognition scores |
|  |  | 2 | Deviant cognition scores (implausibly high) |
|  |  | 6 | Deviant MRI (volumetric outliers, trauma, extreme atrophy) |
|  |  | 29 | Incomplete MRI scanning protocol |
| **Cross-sectional analysis** | 143 |  |  |
|  |  | 52 | Lost to follow-up |
|  |  | 47 | Non-compatible MRI scanning protocol |
|  |  | 7 | Missing cognition scores |
|  |  | 2 | Deviant MRI (volumetric outliers, trauma, extreme atrophy) |
| **Longitudinal analysis** | 35 |  |  |

Supplement E: Results of longitudinal analysis on subset of population (N=35)

Note: 10 patients (28.6%) were scanned on different devices at the two timepoints. Due to missing data, only SDMT scores were included in the longitudinal analysis.

|  | Baseline (Year 0) | Follow-up (Year 3) | P-value* |
| --- | --- | --- | --- |
| SDMT (M ± SD) | 50.29 ± 12.01 | 51.63 ± 13.07 | 0.25 |
| Brainstem (mm^3^, M ± SD) | 23486.43 ± 3053.53 | 23530.76 ± 2996.84 | 0.59 |
| Amygdala (mm^3^, M ± SD) | 3271.02 ± 414.21 | 3253.21 ± 433.44 | 0.46 |
| Hippocampus (mm^3^, M ± SD) | 6641.60 ± 856.45 | 6608.27 ± 857.52 | 0.10 |
| Thalamus (mm^3^, M ± SD) | 11561.68 ± 1594.51 | 11452.55 ± 1512.22 | 0.12 |
| Cortex (mm^3^, M ± SD) | 453864.93 ± 60138.19 | 430217.04 ± 56646.37 | 0.16 |
| GM (mm^3^, M ± SD) | 617342.04 ± 74135.49 | 583278.44 ± 70448.65 | 0.09 |

Table E-1: Brain MRI volumetric measurements of structures relevant for cognition
** Wilcoxon signed rank test*

| Change in: | Correlation with change in SDMT score (correlation coefficient R*) | P-value* |
| --- | --- | --- |
| Brainstem volume | 0.05 | 0.77 |
| Amygdala volume | -0.03 | 0.85 |
| Hippocampus volume | 0.15 | 0.40 |
| Thalamus volume | 0.15 | 0.41 |
| Cortex volume | 0.07 | 0.07 |
| GM volume | 0.05 | 0.05 |

Table E-2: Correlations between changes in volumetric measurements of structures relevant for cognition and changes in SDMT scores over a three-year period
** Spearman correlation*
